# A Survey of Modern Data Acquisition and Analysis Systems for Environmental Risk Monitoring in Aquatic Ecosystems

**DOI:** 10.3390/s26051566

**Published:** 2026-03-02

**Authors:** Nicola Perra, Daniele Giusto, Matteo Anedda

**Affiliations:** Consorzio Nazionale Interuniversitario per le Telecomunicazioni (CNIT), Unità di Ricerca di Cagliari (CNIT UdR Cagliari), Department of Electrical and Electronic Engineering, University of Cagliari, 09123 Cagliari, Italy; n.perra10@studenti.unica.it (N.P.);

**Keywords:** aquatic environmental monitoring, environmental risk assessment, water quality monitoring systems, in situ sensor networks, data acquisition and telemetry, autonomous monitoring platforms, internet of Things (IoT) for environmental monitoring, low-power wide-area networks (LPWAN), artificial intelligence and machine learning, climate change and aquatic ecosystems

## Abstract

This survey is an integrated and complete summary of the strategies and technological systems of surveying environmental hazard in marine, freshwater, and brackish environments. Contrary to the previous articles where the separate parts of the monitoring chain are investigated or certain environments/enabling technologies are considered, the given work has a cross-domain approach that unites sensing modalities, data acquisition schemes, communication schemes, operational platforms, data analytics, energy management schemes, and regulatory compliance into one consistent framework. The survey systematically examines the entire sensing-to-cloud pipeline, which includes sensor technologies, data acquisition systems, telecommunication infrastructures, and a variety of monitoring platforms such as buoy-based systems, Unmanned Surface Vehicles (USVs), Autonomous Underwater Vehicles (AUVs), and Unmanned Aerial Vehicles (UAVs). In addition, it touches on the administration and examination of mass environmental data, including cloud-based systems and AI-based methods of automated feature identification, anomaly recognition and predictive modeling. The key points of the autonomy of the system, including power supply solutions and energy-conscious management, are also mentioned, as well as the relevant regulations on the environmental monitoring nationally, at the European level, and globally. This paper presents a systematic six-step design process of aquatic environmental monitoring systems: (1) risk categorization, (2) physical data acquisition systems, (3) monitoring platforms, (4) data management & analytics, (5) energy autonomy strategies, and (6) regulatory compliance. The systematic framework offers researchers and practitioners practical guidelines to follow when designing end-to-end systems, thus completing the gaps in the historically disjointed research strands and going beyond the traditional domain- and technology-based studies.

## 1. Introduction

Environmental monitoring has become an essential component of contemporary strategies for environmental protection, risk mitigation, and sustainable resource management [1]. Accelerated industrialization, urban growth, climate change, and the intensification of human activities have significantly increased both the frequency and the complexity of environmental hazards, affecting terrestrial, atmospheric, and aquatic systems alike [2]. In this context, the availability of continuous, reliable, and spatially distributed environmental data is critical to support evidence-based decision-making and to enable timely responses to emerging risks.

Aquatic ecosystems, spanning marine, freshwater, and transitional environments, are critical to global biogeochemical cycles, biodiversity preservation, and the sustained delivery of essential ecosystem services [3]. These systems support fisheries, transportation, recreation, and drinking-water supplies, while simultaneously acting as sinks and transport pathways for pollutants originating from land-based and offshore activities. At the same time, aquatic environments are especially vulnerable to a broad range of stressors [4], such as chemical and plastic pollution, eutrophication and harmful algal blooms, overexploitation of biological resources, hydromorphological alterations, and climate-driven processes including ocean warming, acidification, sea-level rise, and changes in hydrological regimes. The cumulative and often synergistic nature of these pressures poses significant challenges to traditional monitoring approaches based on sparse or episodic measurements.

Recent technological advances have profoundly transformed aquatic environmental monitoring [5]. The convergence of miniaturized sensors, low-power embedded electronics, wireless and satellite communications, and cloud-based data infrastructures has enabled the deployment of distributed, autonomous monitoring systems capable of long-term operation in harsh and remote environments. In parallel, the increasing volume and heterogeneity of acquired data have motivated the adoption of advanced data-processing and analysis techniques, including artificial intelligence and machine learning, to extract meaningful patterns, detect anomalies, and support predictive assessments. Nevertheless, the design of effective monitoring architectures remains inherently multidisciplinary and constrained by trade-offs among measurement accuracy, energy autonomy, maintenance requirements, communication range, and system scalability. This paper evaluates current data acquisition and analysis systems for aquatic environmental-risk monitoring, with a primary focus on in situ solutions. Such platforms are vital for accurately capturing the temporal and spatial dynamics that define aquatic ecosystems.

Recent literature on aquatic environmental monitoring tends to focus on specific segments of the end-to-end monitoring chain. Regulatory and program-driven perspectives emphasize monitoring objectives, assessment principles, and harmonization needs under legislative frameworks [6,7]. Technology-oriented reviews often concentrate on individual layers, such as in situ sensing methods for marine analytes [8], autonomous and remote devices for detecting contaminants and plastic debris [9], or IoT-based water monitoring models and taxonomies grounded in real-time data acquisition [10]. Communication-focused surveys address network architectures and emerging wireless technologies for water-quality monitoring, with particular attention to energy efficiency and long-range links [11,12], while platform-centric contributions provide detailed designs and validations of low-cost buoy-based systems [13]. In contrast, this work adopts a risk-driven organizing principle spanning marine, freshwater, and brackish environments, and consolidates the monitoring pipeline from sensing and DAQ to telecommunications, monitoring platforms, data processing, power supply strategies, and regulatory considerations into a single comparative framework. This comparison is summarised in Table 1.

To the best of our knowledge, no existing survey provides a unified and systematic treatment of aquatic environmental-risk monitoring that jointly addresses the full spectrum of technological, architectural, and regulatory aspects across different aquatic domains. Most prior works examine isolated components of the monitoring chain or focus on specific environments, stressors, or enabling technologies. In contrast, this article offers a holistic and cross-domain perspective, integrating sensing modalities, data acquisition architectures, communication strategies, monitoring platforms, data analytics, energy management solutions, and compliance with regulatory frameworks within a single, coherent framework. By bridging these traditionally fragmented research threads, the proposed survey enables readers to develop a more comprehensive understanding of how end-to-end monitoring systems can be designed, deployed, and optimized to effectively support aquatic environmental risk assessment and management.

To address the complexity of modern aquatic monitoring, thissurvey goes beyond conventional state-of-the-art review and introduces a rigorous six-step operational procedure designed to guide the development of end-to-end monitoring systems. This process integrates: (i) environmental risk identification, (ii) sensing modality selection, (iii) platform and acquisition strategy definition, (iv) communication infrastructure design, (v) AI-driven data analytics, and (vi) regulatory compliance verification. This structure shifts the focus from isolated technological choices to a cohesive system-engineering perspective. Nonetheless, our synthesis indicates that fully autonomous and reliable aquatic monitoring remains constrained by structural gaps, including lack of quantitative uncertainty measurement, the limited practical generalizability of artificial intelligence systems, the trade-offs between energy autonomy and telemetry requirements in mobile systems, and the ongoing fragmentation between international regulations and technical implementations. By anticipating such gaps early, this survey aims both to offer a taxonomy of existing solutions, and to provide a strategic roadmap for overcoming the operation bottlenecks that currently limit the widespread usage of advanced aquatic monitoring technologies.

The rest of the paper is organized as follows: Section 3 reviews the principal environmental risks affecting marine, freshwater, and brackish ecosystems, providing a conceptual classification of stressors and observable alterations. Section 4 focuses on physical data acquisition systems, including sensor technologies, data acquisition boards, and telecommunication solutions, highlighting their role and limitations in aquatic monitoring. Section 5 addresses data management and analysis, with emphasis on cloud-based infrastructures and artificial intelligence techniques. Section 6 examines the main monitoring platforms, ranging from buoy-based systems to unmanned surface, underwater, and aerial vehicles. Section 7 and Section 8 discuss energy autonomy and regulatory aspects relevant to long-term environmental monitoring, respectively. Finally, Section 9 summarizes the main conclusions and outlines future research directions.

## 2. Methodology

This survey is based on a thematic and problem-driven exploration of the scientific literature, initially motivated by aquatic environmental-risk monitoring applications but not limited to domain-specific studies. The review process followed the same topic structure adopted in the manuscript, with literature being identified and integrated independently for each major technological and methodological area. For each topic, relevant studies were primarily identified through academic literature search engines using simple keyword-based searches reflecting the specific technology or system component under analysis. While aquatic monitoring applications guided the overall scope of the survey, the literature search often extended to more general technological domains when domain-specific studies were scarce or insufficient. Search terms evolved naturally during the review process as additional concepts, terminology, and related technologies emerged from the examined literature. The selected studies were read in full and interpreted with respect to their relevance to the specific topic addressed in each section, and subsequently contextualized within aquatic monitoring scenarios when applicable. This iterative approach allowed the literature review and the writing process to proceed in parallel, supporting a coherent integration of heterogeneous technological contributions while maintaining a consistent application-oriented perspective.

### Literature Search Strategy

The literature search underlying this survey was conducted using a structured, theme-oriented strategy aligned with the technical scope and organization of the manuscript. Widely used academic search engines were employed as primary discovery tools, including Google Scholar and PubMed, while full texts were mainly retrieved through major scientific publishing platforms such as MDPI, IEEE Xplore, and Elsevier. Additional sources, including technical reports, standards, manuals, and hardware specification sheets, were consulted when relevant to the analysis of system architectures or enabling technologies. The review primarily focused on literature published within the last fifteen years. Earlier works were selectively included when they provided foundational concepts, reference architectures, or historical context necessary to understand the evolution of specific technologies. Within this temporal window, priority was given to studies closely aligned with the topic addressed in each section, followed by highly cited contributions and more recent publications, reflecting both relevance and impact within the field. Search queries were formulated independently for each thematic area of the survey and evolved iteratively as the review progressed. Keyword combinations reflected both application-oriented and technology-oriented perspectives and included, among others:

- Water quality monitoring;
- Aquatic environmental monitoring;
- Optical and electrochemical sensors;
- Data acquisition systems;
- Embedded monitoring platforms;
- LPWAN technologies (e.g., LoRa, NB-IoT);
- Satellite machine-to-machine communications;
- Autonomous monitoring platforms (buoys, USVs, UUVs, UAVs);
- Energy autonomy;
- Power management;
- Environmental data analytics;
- Machine learning for environmental monitoring.

This approach allowed the identification of relevant contributions even when specific technologies were predominantly discussed outside aquatic-focused studies. Inclusion criteria comprised peer-reviewed journal articles and conference papers written in English and addressing aquatic environmental monitoring applications or technologies transferable to such contexts. Studies were selected based on their relevance to system-level design, sensing strategies, communication architectures, operational constraints, or data analysis methodologies. Exclusion criteria included works with a purely theoretical focus lacking practical implications for monitoring systems, as well as application studies unrelated to environmental sensing and without clear technological transferability. Over a period of approximately six months, a comprehensive review of roughly one thousand documents was conducted, including peer-reviewed articles, conference proceedings, technical reports, and data sheets. Applying the selection criteria previously described led to the curation of over two hundred sources, which are formally cited in the bibliography and analyzed throughout this work.

## 3. Environmental Risks in Aquatic Ecosystems

In recent decades, while the global importance of aquatic ecosystems is well established, these environments have faced escalating anthropogenic pressures. Regardless of their classification as marine, freshwater, or transitional systems, such habitats are exposed to comparable human-induced stressors resulting from both direct and indirect activities. Continuous monitoring is therefore essential for the effective management and mitigation of these environmental risks. In this work, environmental stressors affecting aquatic ecosystems are organized according to both water type (marine, freshwater, and brackish) and dominant stressor categories. Specifically, the analysis focuses on pollution-related stressors, resource exploitation, and climate-induced pressures.

### 3.1. Marine Risks

Pollution remains one of the most prominent and extensively documented risks affecting marine ecosystems. Among pollution-related stressors, microplastics have attracted increasing scientific and public attention due to their widespread occurrence and persistence in marine environments. Gola et al. [14] report that microplastic contamination in aquatic systems is attributable to anthropogenic activities and is particularly pronounced in proximity to urbanized areas, affecting both water bodies and marine organisms. Owing to their high dispersal potential, microplastics have also been detected in remote marine regions. For instance, Obbard et al. [15] identified microplastic traces in ice cores collected during Arctic expeditions, highlighting the global extent of this form of pollution.

Microplastics are commonly distinguished into primary particles, intentionally manufactured at small sizes, and secondary particles generated through the fragmentation of larger plastic debris [16]. While microplastics represent only a fraction of the overall plastic load in the oceans, Eriksen et al. [17] estimate that at least 5.25 trillion plastic particles float at the ocean surface, underscoring the scale of plastic pollution. From a monitoring perspective, the coexistence of macro- and microplastics implies fundamentally different observational challenges, driven primarily by their contrasting spatial scales and physical characteristics.

In addition to plastic pollution, hydrocarbon spills constitute one of the most widely recognized forms of marine contamination. The environmental impacts of oil spills have been studied for decades, beginning with early assessments such as the analysis of the Torrey Canyon disaster by Griffith et al. [18]. Despite progressively stricter regulations [19], large-scale accidents have continued to occur, exemplified by the Deepwater Horizon spill in 2010 [20]. Oil pollution affects marine ecosystems through multiple mechanisms, including physical smothering, chemical toxicity, community-level alterations, and habitat loss [21]. The nature and severity of these impacts depend strongly on oil type, with less-refined fuels posing significant smothering hazards and more refined products exhibiting higher acute toxicity.

Another critical stressor is eutrophication, defined as an excessive enrichment of water bodies with nutrients, primarily nitrogen and phosphorus, that promotes harmful algal blooms (HABs) and disrupts trophic dynamics [22]. Nutrient inputs largely originate from agricultural runoff, sewage discharges, industrial effluents, and riverine transport [23,24]. HABs can induce hypoxic conditions and compromise ecosystem health [25]. As the prevention of bloom formation is generally more effective than remediation, continuous monitoring of nutrient concentrations and oxygen dynamics is essential.

Marine ecosystems are also affected by underwater noise pollution, which represents an indirect but increasingly recognized anthropogenic stressor. The World Health Organization identifies noise pollution as a major environmental hazard [26]. Hou et al. [27] reported that elevated noise levels negatively influence fish reproductive cycles and biomass, primarily by increasing energetic stress. Peng et al. [28] further emphasize that the ecological effects of underwater noise remain incompletely understood, owing to variability in sound sources and species-specific sensitivities. According to ISO standards [29], underwater sound can be described in terms of sound pressure and particle motion, each representing distinct physical quantities with biological relevance.

Beyond pollution, marine ecosystems are subject to exploitation-related pressures. Overfishing refers to harvesting marine organisms at rates exceeding their reproductive capacity [30]. Pauly [31] documented the phenomenon of “fishing down the food web,” whereby fisheries progressively target lower trophic levels, altering ecosystem structure and resilience. Monitoring fishing pressure and stock dynamics is therefore essential for sustainable resource management.

Climate change constitutes a pervasive and long-term driver of marine ecosystem stress. It manifests through ocean warming, acidification, and sea-level rise. According to the State of the Global Ocean report, global mean sea surface temperature increased at a rate of 0.13 ± 0.01 °C per decade between 1982 and 2023 [32]. Satellite altimetry indicates that the global mean sea level has risen by more than 10 cm over the past three decades, with an accelerating trend [32]. Concurrently, ocean acidification has intensified, with pH decreasing by approximately 0.017 units per decade since the mid-1980s [32,33]. These processes have far-reaching implications for marine habitats, species distributions, and biodiversity [34,35,36,37].

### 3.2. Freshwater Risks (Rivers and Lakes)

While marine ecosystems receive substantial attention, many anthropogenic stressors originate on land and primarily affect freshwater systems. Rivers, lakes, and reservoirs are exposed to industrial and agricultural effluents, emerging micropollutants, and hydromorphological alterations, while also being vulnerable to climate-driven changes in hydrological regimes.

Agricultural activities account for approximately 70% of global freshwater use and represent a major source of nutrient pollution. Runoff enriched with nitrogen and phosphorus contributes to eutrophication [38]. Industrial discharges further degrade water quality, as documented by Lemessa et al. [39], who observed significant deterioration downstream of an industrial park in Addis Abeba. Similar patterns were reported for the Po River by Panepinto et al. [40], highlighting cumulative impacts from urban and industrial sources. In recent years, attention has expanded to emerging contaminants, including pharmaceutical compounds detected in urban wastewater and groundwater [41].

Human interventions such as dam construction and channelization also exert profound impacts on freshwater ecosystems. Dams alter flow regimes, sediment transport, temperature profiles, and migratory pathways [42]. Ely et al. [43] demonstrated significant hydrological alterations in the Upper Paraguay River basin, underscoring the need to balance water resource management with ecosystem preservation. Channelization further disrupts river–floodplain connectivity and biodiversity, as shown by Kennedy et al. [44].

Climate change exacerbates these pressures by altering precipitation patterns and hydrological extremes. The IPCC reports with high confidence that human influence has increased the frequency and intensity of heavy precipitation events in some regions, while enhancing drought severity in others [45]. These changes directly affect freshwater availability, flood risk, and ecosystem stability.

### 3.3. Brackish Water Risks (Lagoons and Estuaries)

Situated at the transition between marine and freshwater systems, brackish lagoons and estuaries act as integrative interfaces that accumulate and mediate stressors originating from both environments. According to Kennish [46], the principal anthropogenic pressures affecting these ecosystems include habitat degradation and loss, excessive nutrient inputs, overfishing, and chemical contamination, together with freshwater diversion and the introduction of invasive species.

Recent studies illustrate the fragility of these systems. Derrien et al. [47] analyzed a coastal lagoon in central Chile, revealing recurrent contamination and episodic degradation linked to sediment remobilization. Rodgers et al. [48] identified localized hotspots of potentially toxic elements in the Clyde River estuary, while Yi et al. [49] demonstrated strong interactions between heavy metal contamination and microbial community structure in the Yangtze River estuary.

Despite their ecological importance, estuarine systems remain underrepresented in climate-change research. Biguino et al. [50] reported that only 0.11% of climate change studies up to 2022 focused on estuarine environments with in situ observations, highlighting a substantial knowledge gap. This scarcity of data poses challenges for the design and validation of monitoring systems in brackish waters.

Overall, the stressors discussed in this section underscore the diversity and complexity of pressures acting on aquatic ecosystems. By outlining the principal environmental stressors affecting aquatic ecosystems, this section establishes the contextual framework required to motivate the selection of sensing, data-acquisition, and communication technologies discussed in the following sections. The classification presented in Table 2 provides a conceptual framework that organizes environmental stressors according to the nature of the observable alterations they induce in aquatic systems.

### 3.4. From Risk Categories to Monitoring Requirements

The following part translates the risk types as taken into consideration in the aquatic environmental monitoring into indicative monitoring requirements, i.e., a small set of quantifiable values that (i) follow the progress of any stressor and (ii) aid in risk-based management decisions. The mapping should be localized to meet local goals, sensor accessibility and practical considerations. A core set of indicators used in freshwater and marine systems to assess the condition of nutrient enrichment, eutrophication, and harmful algal blooms includes chlorophyll-*a* (or fluorescence proxies), dissolved and total nitrogen and phosphorus species, dissolved oxygen, and turbidity or total suspended solids. High-frequency nutrient sensing is commonly used to measure rapid variability of loads and antecedent conditions [51].

Other parameters, such as water transparency and a cyanobacteria-specific optical proxy can be included in lacustrine systems but salinity-dependent optics and benthic-pelagic interactions require measures in estuarine and lagoonal systems. Remote sensing is often used to delineate bloom-related gradients [52,53].

Core indicators for industrial and agricultural effluents typically include nutrients, turbidity or total suspended solids, and, in some cases, organic-load proxies such as chemical oxygen demand (COD) or biological oxygen demand (BOD). These measurements are often complemented by composite indices and spatial analytics to pinpoint hotspots [54,55]. When agrochemicals are a concern, monitoring specifications are extended to include target analytes (e.g., selected pesticides) in the water column and, where appropriate, in sediments; results are then interpreted in light of contextual hydrological information, such as discharge [56]. For micro- and nanoplastic monitoring, metrics such as concentration (number of particles per unit volume), size-distribution characteristics and, where feasible, identification of polymer type or material class are used as baseline measures, typically based on surface sampling techniques (e.g., manta trawls or in situ pumping [57]). Macroplastic and floating-litter monitoring typically focuses on parameters such as surface density, abundance, mass (or associated proxies), and spatial accumulation patterns, often using multi-platform survey data at the basin scale [58]. During oil and chemical spill events, rapid-response monitoring is based on hydrocarbon-based fluorescence or polycyclic aromatic hydrocarbon (PAH)-related signals in combination with measurements of slick length and depth, where active sensors like laser induced fluorescence (LIF) LiDAR are one possible tool to identify the presence and thickness of a slick [59,60]. In ocean warming research [61,62], important observables include sea surface temperature, subsurface thermal stratification (including thermocline depth), marine heatwave indices, and integrated heat content proxies. The temperature observations are typically interpreted jointly with pH and salinity in estuarine and other transitional brackish environments to distinguish between marine intrusion and freshwater inputs [63], and the temperature–salinity structure (or density gradients) together with dissolved oxygen measurements can be used to identify hypoxic events caused by stratification [64].

In the measurement of sea level rise, both tide gauge and satellite altimetry measurements are integrated with vertical land motion measurements (GNSS/GPS), and (where salinity intrusion is relevant) groundwater measurements and salinity measurements are used [65,66]. In estuaries, discharge and salinity-based metrics of intrusion (e.g., the position of the salt-wage or longitudinal salinity profiles) [67], are synthesised using sea level forcing to investigate prospective changes. In addition, temporally resolved salinity and temperature recordsare interpreted with respect to hydroclimatic drivers (e.g., rainfall or river discharge) form a central modernisation in climate attribution research [68]. Ocean acidification monitoring is often performed by concomitant pH and carbonate system measurements (along with temperature and salinity measurements) to enable full interpretation of carbonate system proxies, including alkalinity or saturation state proxies [69].

Underwater noise pollution also involves the monitoring of ambient sound pressure level (SPL), impulsive source sound exposure level (SEL) or peak pressure and long-term passive acoustic trends; acoustic indices are used to quantify the soundscape composition [70,71].

Streamflow measurements, groundwater indicators, and drought indices measure the extent and length of water shortages during drought or low flow events [72,73] and flood monitoring emphasises discharge peaks, timing and suspended sediment proxies and hazard characterisation is supported by exceedance-based measures [74,75]. When monitoring hydraulic infrastructure (e.g., dams and channelisation schemes), key metrics include flow-regime alteration, sediment transport and loading, and retention-sensitive water-quality variables (e.g., oxygen dynamics and nutrient transformations) [76,77].

Emerging contaminant monitoring integrates target concentration measurements, including pharmaceuticals, per- and polyfluoroalkyl substances (PFAS), and pesticides, with time-integrated or passive monitoring methods, where justified, effect-based endpoint indicators (e.g., bioassays or biomarkers) can also be included to capture integrated biological effects [78,79]. Lastly, measures of overfishing and fishery stress are commonly evaluated in terms of catch-per-unit-effort (CPUE) and standardised indices of abundance or biomass, which are interpreted relative to literature-based reference points, where available [80,81]. Table 3 provides an example of a risk-based synthesis: different categories of environmental stressors (e.g., plastic pollution, oil spills, harmful algal blooms, effluents, climate-driven effects, and brackish multi-stressor interactions) are mapped to monitoring-system configurations along the sensing-to-cloud continuum. Category-specific requirements may include high-rate and burst-mode data capture for rapidly evolving phenomena (e.g., spills), low-power duty-cycled operation with LPWAN backhaul for persistent HAB monitoring, and hybrid satellite–cellular connectivity for remote climate-observing arrays. These design choices are tailored to the observable parameters (e.g., hydrocarbon fluorescence, chlorophyll-*a*) while exposing trade-offs in spatiotemporal resolution, energy autonomy, and platform mobility (e.g., moored buoys versus USVs), ultimately supporting robust and regulation-compliant risk assessment.

## 4. Physical Data Acquisition Systems for Aquatic Monitoring

The preceding sections outlined the principal types of aquatic ecosystems and the array of problems they face, from pollution to other anthropogenic stressors. For each case, the need to control these pressures emerged clearly—both to prevent their onset and to manage conditions already underway. As summarized in Table 2, each environmental stressor discussed can be associated with one or more physical, chemical, or biological quantities that are relevant for monitoring purposes. In situ monitoring is particularly important, as it enables the continuous observation of key parameters directly within the system of interest. Compared with traditional laboratory analyses, these measurements allow for the real-time tracking of ecosystem conditions, thereby facilitating early anomaly detection and more effective management of ongoing environmental issues [82].

**Figure 1:**
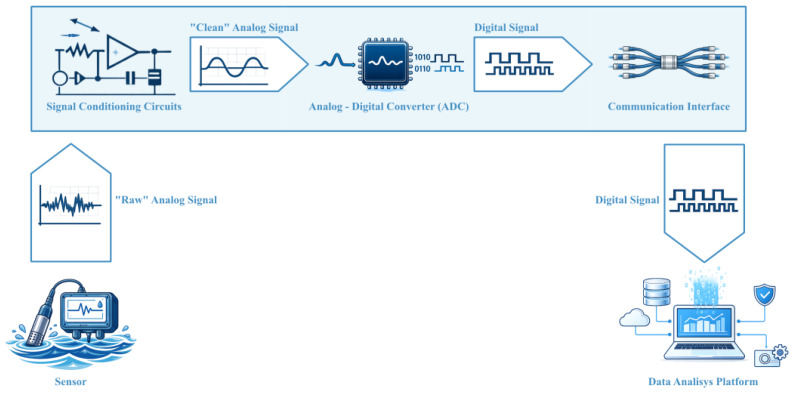
The signal pathway: From the sensor, through the data acquisition board, to the Data Analysis Platform.

In aquatic environments, these requirements are further constrained by long-term autonomy, exposure to harsh operating conditions, and limited physical accessibility, which place additional demands on monitoring hardware and system architecture. These considerations introduce the topic of data acquisition systems, which constitute the technological foundation of modern environmental monitoring and whose data path, from the sensor to the data analysis platform, is illustrated in Figure 1.

### 4.1. Introduction to Acquisition Systems

Any measurement performed in an aquatic environment relies on an acquisition infrastructure capable of converting physical, chemical, or biological quantities into digital information. Such an infrastructure typically comprises sensors, signal-conditioning circuits, and data-acquisition hardware, which operate as a coordinated chain to ensure signal integrity and usability.

Sensors (i.e., transducers) convert the quantity to be measured into a proportional electrical signal. A basic classification distinguishes between passive and active sensors based on whether they require an external power source to perform their sensing function. Passive sensors operate without an external supply and draw the energy required for signal generation from the system being measured, whereas active sensors include a power source and do not exploit the measurand itself to perform the measurement [83]. Because the sensor output represents an analogue of the measured quantity, it may appear as a voltage on the order of millivolts or nanovolts, a current, or another electrical parameter; in some cases, it may share the measurand’s frequency or be carried on a carrier modulated by the measurand [83].

The sensor output is subsequently routed to signal-conditioning circuits, whose purpose is to render the signal compatible with the requirements of the data-acquisition hardware. Signal-conditioning stages typically provide filtering, amplification, linearization, isolation, and sensor excitation. The conditioned signal is then processed by the data acquisition hardware, converted to digital form via an analogue-to-digital converter (ADC), and transferred to a computing system for visualization, storage, and analysis [84].

### 4.2. Environmental Monitoring Sensors

According to ANSI standard MC6.1 (American National Standards Institute), a sensor (or transducer) is “a device which provides a usable output in response to a specific measurand” [85]. Beyond this formal definition, sensors are characterized by a set of performance parameters that determine their suitability for long-term environmental monitoring.

Among these parameters, accuracy and precision are particularly relevant. Accuracy denotes the relationship between the value reported by the sensor and the actual value obtained using a reference method, whereas precision describes the reproducibility of repeated measurements under unchanged conditions [86]. If the output of a sensor varies over time while the input remains constant, this behavior is referred to as drift. Sensor accuracy is typically evaluated through static calibration procedures, in which all inputs are held constant except for the measurand under investigation, which is varied slowly across the measurement range. The resulting calibration curve establishes the relationship between the sensor output and the applied input; its slope corresponds to the sensor sensitivity, defined as the ratio between a variation in the output signal and the associated variation in the input quantity at a specified operating point [87].

Linearity describes the deviation of the calibration curve from a specified straight-line behavior, whereas resolution corresponds to the smallest change in input that produces a detectable change in output. The measurement range is defined as the interval between the minimum and maximum measurable values [87]. As specified by IUPAC, the response time is the “time which elapses, when there is a stepwise change in the quantity to be measured, between the moment when this change is produced and the moment when the indication reaches a value conventionally fixed at 90% of the final change in indication” [88].

Beyond the passive/active distinction, sensors may be classified according to the measurand (physical, chemical, or biological), the transduction mechanism (optical, mechanical, pneumatic, electrical), or the application domain (environmental, industrial, biochemical, pharmaceutical). With respect to the measured quantity, physical sensors provide information on physical properties of the system, chemical sensors convert chemical inputs into analytically useful signals related to the presence or concentration of a species, and biological sensors respond to biological entities or processes [86].

A detailed overview of the principal sensor classes employed in marine and coastal monitoring—including commercial instruments and research-stage solutions—is provided by Briciu-Burghina et al. (2023) [89]. The advancement of in situ aquatic monitoring is also increasingly reliant on the deployment of multiparameter sondes, which allow for the simultaneous acquisition of diverse physicochemical and optical data. These devices integrate a wide array of sensors to characterize water bodies: standard configurations typically employ electrochemical and physical sensors for measuring temperature, salinity, conductivity, dissolved oxygen, and pH [90]. Furthermore, modern sondes incorporate advanced optical sensors, specifically utilizing UV–Vis absorption spectrometry and fluorescence-based probes. These optical components enable the high-resolution detection of specific indicators such as nitrates, Chemical Oxygen Demand (COD), turbidity, and chlorophyll-*a* [91]. The primary advantage of these integrated systems lies in their ability to provide a holistic view of the ecosystem’s health, mitigating the limitations of discrete sampling.

Table 3 outlines the recurring indicators derived from the summary of aquatic risk parameters presented in Table 4, providing representative examples of the technical specifications for commercial in situ monitoring probes. While it is inherently difficult to compare sensors designed for disparate parameters, a comprehensive evaluation remains elusive even for cross-cutting metrics such as power consumption or calibration intervals. As evidenced in Table 4 and the referenced datasheets, manufacturers often fail to report specifications systematically or consistently across their product lines even within the same organization.

With regard to this, ISO 22013:2021 [92] provides a sensor-agnostic framework for specifying and reporting marine sensor performance, including accuracy assessed against a reference through bias and reproducibility, and a recommended public data sheet structure that also reports operational parameters such as sample rate and operating power consumption. In particular, when bias is statistically insignificant, ISO 22013 defines the sensor accuracy as the reproducibility standard deviation. Beyond these reporting requirements, system-level design typically requires co-optimizing sensing, acquisition, and communications under an energy budget: higher sampling rates and higher channel counts increase payload generation, which in turn constrains the telemetry strategy and duty cycle. Communication energy per delivered bit depends on the link budget and selected physical layer; therefore, for constrained long-range links, local aggregation (e.g., averaging, feature extraction) and event-driven reporting are often adopted to reduce transmitted volume. Accordingly, ISO 22013-style specifications can be used as a common baseline to compare accuracy–power–data volume trade-offs across heterogeneous sensor families, while acknowledging that the optimal operating point is application- and platform-dependent. For a widely used class of in situ multiparameter sondes, pH is typically specified at ±0.1 pH units near the calibration temperature (degrading to ±0.2 pH units over wider temperature excursions), while dissolved oxygen (DO) is commonly reported as ±1% of reading up to ∼200% air saturation and ±5% at supersaturated regimes; optical turbidity may be reported as 0.3 FNU or ±2% of reading in the low-to-mid range, and pressure/level sensors are typically within ∼0.03–0.04% of full scale [93]. ISO 22013 conceptualizes accuracy as a combination of trueness (bias) and precision and provides an illustrative data-sheet example for sound speed sensors (e.g., accuracy ±0.019 m/s and repeatability ±0.009 m/s). Importantly, these figures describe single observations under stated conditions: time/space averaging reduces the contribution of random noise approximately with $1/\sqrt{N}$ for independent samples, but it does not mitigate systematic components such as bias, drift, and biofouling, which often dominate long deployments. The metrological implications of traceability, calibration chains, and data acceptance criteria are discussed further in Section 8.

**Table 4:** Technical specifications of state-of-the-art aquatic sensors for environmental monitoring.

Parameter	Sensor Model	Relative Accuracy	Typical Power Draw	Calibration Cycle
Turbidity	Seabird AC-S [94]	$\pm 0.01\;m^{-1}$	0.37A @ 12VDC	2-year cycle
CTD (Cond., Temp., Depth)	Seabird 19plus V2 [95]	±0.002 °C (−5 to +35 °C); ±0.01 °C (+35 to +45 °C)	9–28VDC, current on request	Undeclared
Nutrients (N, P)	Seabird DeepSuna [96]	Greater of 10% of reading	0.625A at 12V	900h
Chlorophyll-a	Valeport Hyperion [97]	Undeclared	<600 mW at 9–28VDC	Undeclared
Dissolved Oxygen	Seabird SBE-43 [98]	±2% of saturation	6.5–24VDC; 60mW	>1000 h
pH	Seabird SeaFet V2 [99]	±0.05pH	340–400mW	Undeclared
CO_2_	CONTROS HydroC™ CO_2_ [100]	±0.5% of reading	300mA @ 12V	Undeclared

### 4.3. The Data Acquisition Board (DAQ)

Regardless of the measurand and sensing principle employed, sensors generate analogue signals that must be processed before they can be stored, transmitted, or analyzed. In environmental monitoring systems, the data acquisition board (DAQ) acts as the interface between the physical world and the digital domain, and its characteristics directly influence data quality, system reliability, and energy consumption.

The first processing stage is signal conditioning, whose role is to transform the sensor’s raw output into a form compatible with the input requirements of an analogue-to-digital converter. Signal-conditioning circuits typically perform amplification, level shifting, filtering, impedance matching, modulation, or demodulation [87]. Analogue-to-digital converters then generate a digital representation of the signal, commonly using sample-and-hold circuitry for sampling and quantization stages for digitization. Because DAQ systems often accept multiple analogue inputs while providing a limited number of ADC channels, multiplexers are frequently employed to sequentially connect individual inputs to a shared converter [83].

From an architectural perspective, DAQ solutions can be broadly divided into host-based systems and embedded platforms. Commercially available options include PCI-Express boards installed in personal computers, proprietary modular systems, standalone instruments, and embedded platforms based on system-on-chip (SoC) or FPGA architectures [101]. In all cases, digitized data must be transferred to a destination for processing or storage via a communication interface, which encompasses both the physical medium and the communication protocol shared by transmitter and receiver [84]. Common interfaces include PCIe, USB, Ethernet, and serial standards such as UART, SPI, I^2^C, RS-232, and RS-485 [102,103,104]. Multiple interfaces are often implemented within a single system to enhance flexibility and redundancy.

Beyond laboratory or industrial settings, in situ monitoring of aquatic environments prioritizes compact, low-power solutions capable of supporting distributed and long-term deployments. These requirements often entail trade-offs in measurement accuracy and system complexity. To satisfy these requirements, data acquisition is typically implemented using microcontroller-based systems equipped with either integrated or external analog-to-digital converters (ADCs), commonly referred to as data loggers [105]. Hercog et al. present a PCB-based solution, SimpleDAQ, built around a Microchip PIC18F27J53 microcontroller, enabling 12-bit ADC acquisition but constrained by the limited input range imposed by the absence of a dedicated analogue front end [106]. Wickert et al. propose the open-source ALog platform, based on Arduino, which emphasizes compactness, low energy consumption, and affordability while providing a dedicated 16-bit ADC and multiple communication options [107]. More advanced yet still energy-efficient solutions based on single-board computers are also available; for example, the MCC-118 HAT offers eight 12-bit analogue inputs and can be stacked on Raspberry Pi platforms to increase channel count [108].

### 4.4. Telecommunication Systems

Environmental monitoring relies critically on the reliable transfer of data acquired by sensing nodes to processing or storage units. Telecommunication systems therefore play a key enabling role, supporting automatic data exchange among devices according to the machine-to-machine (M2M) paradigm. Depending on transmission distance, data volume, energy availability, and operational constraints, current solutions can be broadly classified into short-range and long-range communication technologies, a distinction that is particularly relevant for distributed and autonomous monitoring deployments.

#### 4.4.1. Short-Range Communication Technologies

Short-range technologies support local data exchange within monitoring platforms or between closely spaced nodes. The main solutions include Wi-Fi, Bluetooth, and ZigBee. Wi-Fi, standardized under the IEEE 802.11 family, offers high data rates and is suitable where local infrastructure is available. Coverage and throughput are strongly coupled to operating frequency: lower frequencies improve range at the expense of data rate. For instance, IEEE 802.11ah operates below 1 GHz, achieving outdoor ranges up to about 1 km with maximum data rates of approximately 78 Mbps [109], whereas IEEE 802.11n at 2.4 GHz supports up to 600 Mbps and IEEE 802.11be up to 46 Gbps [110]. Coverage further depends on antenna design, propagation conditions, and modulation and coding schemes, with lower data rates providing higher robustness [111]. Experimental evaluations confirm that higher frequencies and wider bandwidths lead to capacity degradation over distance, even when connectivity is preserved [112]. Consequently, Wi-Fi-based solutions are energetically inefficient and poorly suited to remote or long-term autonomous aquatic monitoring.

Bluetooth is characterized by robustness, low power consumption, and low cost. It supports a Basic Rate (BR), offering up to 721.2 kbps (or 2.1 Mbps with Enhanced Data Rate), and Low Energy (LE), optimized for reduced energy consumption while maintaining comparable throughput [113]. However, its limited range, typically on the order of tens of meters, restricts its applicability to local interactions rather than wide-area monitoring.

ZigBee, based on IEEE 802.15.4, provides very low power consumption and supports star, tree, and mesh topologies [114]. It offers data rates up to 250 kbps, with indoor ranges around 60 m and outdoor line-of-sight distances up to approximately 1200 m [115]. While ZigBee and IEEE 802.11ah can achieve similar ranges, ZigBee’s lower throughput limits its suitability for applications requiring higher data rates or low latency. Comparative studies show ZigBee excelling in energy efficiency, whereas IEEE 802.11ah provides superior throughput and reduced association latency for IoT-oriented use cases [116].

#### 4.4.2. Long-Range Communication Technologies

Long-range communications are required when monitoring nodes are distributed over large areas or deployed in infrastructure-less environments. Satellite systems and low-power wide-area networks (LPWANs) represent two fundamentally different approaches.

Satellite communications provide near-global coverage and are categorized into geostationary (GEO) and non-geostationary orbits, including low Earth orbit (LEO) and medium Earth orbit (MEO). GEO satellites offer wide coverage with minimal spacecraft, while LEO constellations provide reduced latency and higher aggregate capacity [117]. Commercial LEO-based services support Machine to Machine communication M2M) and Short Burst Data (SBD) transmissions suited to low-volume environmental monitoring, with payloads limited to 340 B per uplink message (from device to satellite network) and 270 B per downlink message (from satellite network to device) [118]. High-throughput satellite-Internet services, such as Starlink, offer downlink rates of 25–200 Mbps with latencies of 25–100 ms [119], but at the cost of increased energy consumption, hardware complexity, and continuous link management, limiting their suitability for low-power autonomous monitoring stations.

Sigfox operates in sub-GHz ISM bands using ultra-narrowband modulation and supports payloads of 0–12 B at data rates between 100 and 600 bit/s. The network is entirely operator-managed and accessed through subscription-based services. While this simplifies deployment at the device level, it limits user control over infrastructure evolution and availability. Sigfox achieves wide-area coverage with typical communication ranges of approximately 10–40 km, depending on environmental conditions [120,121,122], trading extremely low throughput for extended range and low device energy consumption.

LoRa employs Chirp Spread Spectrum modulation, with performance governed by the Spread Factor, Bandwidth, and Coding Rate. Combined with the LoRaWAN protocol, it supports long-range communication under stringent power constraints and allows users to deploy and manage their own infrastructure [123,124]. Experimental results indicate strong sensitivity to parameter configuration and environmental conditions: for example, throughput as low as 7.80 bit/s was observed for 51-byte payloads using Spread Factor 7 (SF7), while packet reception rates of approximately 40% were measured at 3400 m using Spread Factor 12 (SF12), both in urban settings [125]. These characteristics make LoRa/LoRaWAN well suited to sporadic, low-payload, energy-constrained monitoring applications.

Cellular networks constitute a general-purpose wide-area solution, supporting both low-rate M2M traffic and high-throughput data streams. Operated on provider-managed infrastructure and regulated by the ITU and 3GPP [126], cellular systems offer extensive coverage in populated areas, with global population coverage of approximately 96% for 3G, 93% for 4G, and 55% for 5G [127]. However, the subscription-based model limits infrastructure control and introduces long-term cost and service-dependency considerations.

To support low-power IoT devices, 3GPP has introduced Narrowband-IoT (NB-IoT), which offers data rates up to 200 kbps [128], enhanced coverage, and battery lifetimes reported up to approximately 10 years under constrained transmission schedules [129,130]. More recently, Ambient-IoT (A-IoT) has emerged in 3GPP’s Releases 18 and 19 as a prospective framework for ultra-low-power, potentially battery-less devices powered by energy harvesting [131,132]. As A-IoT is still under development, its role in environmental monitoring remains exploratory.

Duty cycle is a dimensionless indicator that quantifies the fraction of an observation interval during which a radio resource is occupied by transmissions, and it can be expressed as $DC\left[\%\right]=\frac{T_{\mathrm{on}}}{T_{\mathrm{obs}}}\;\cdot \;100$ [133]. In practice, $T_{\mathrm{on}}$ is the cumulative *time-on-air* (ToA) of all packets, while $T_{\mathrm{obs}}$ is the reference time window over which channel occupancy is evaluated [134]. In unlicensed bands, duty-cycle constraints are frequently introduced to promote fair coexistence and to limit aggregate interference, particularly for sub-GHz short-range devices [135]. From a system-design perspective, the *operational* duty cycle is application-driven and depends on message size, physical-layer bit rate, retransmissions, and the selected reporting interval. Therefore, duty cycle directly influences network capacity and, for battery-powered devices, it is a first-order determinant of average power consumption and expected lifetime.

Table 5 highlights a core system-level trade-off: higher-information monitoring (i.e., transmitting raw waveforms, dense time series, or images with minimal on-node reduction) tends to require higher-throughput links, which in turn generally implies higher instantaneous power draw, as exemplified by Wi-Fi (high current, large data rate, short range) and satellite Internet (very high current, large data rate, global coverage). Conversely, ultra/very-low-power radios such as Bluetooth LE, ZigBee, LoRa/LoRaWAN, Sigfox, and Ambient-IoT support low-duty-cycle telemetry with very small to small data rates; this typically pushes the design toward local aggregation/feature extraction (potentially reducing temporal granularity or “measurement fidelity” at the cloud) to remain within payload and energy budgets. Range and data rate are coupled: long-range LPWAN options trade throughput for link budget (very small rates for LoRa/Sigfox/Ambient-IoT), while cellular NB-IoT offers a middle ground (small data rate with variable current draw). From an energy-per-bit perspective, low-rate long-range links often minimize peak current but may increase radio “on-air time” per delivered payload, whereas high-throughput links draw more current but can amortize overhead by transferring large volumes quickly; therefore, the energy-optimal choice depends strongly on whether traffic is sparse status packets (favoring LPWAN/Ambient-IoT/SBD) or bulk data (favoring Wi-Fi or satellite Internet).

**Table 5:** Comparison of telecommunication technologies for aquatic environmental monitoring.

Technology	Typical Range	Typical Current Draw	Average Data Rate
Wi-Fi (802.11)	Short (10–60 m), up to ∼1 km (11ah)	High (100–350 mA) [128]	Large (78 Mbps/46 Gbps) [110]
Bluetooth LE	Very short (10–50 m)	Very Low (∼30 μA) [136]	Moderate (721.2 kbps/2.1 Mbps) [113]
ZigBee (802.15.4)	Short–medium (up to ∼1 km LOS)	Very Low (1–10 mA) [137]	Very small/Small (20–250 kbps) [138]
LoRa/LoRaWAN	Long (km-scale, config.-dependent)	Very low (1–10 mA) [137]	Very small (<10 kbps) [128]
Sigfox	Long (10–40 km typical)	Very low (15–54 mA) [139]	Very small (∼100–600 bps) [128]
NB-IoT	Long (operator-dependent)	Mixed (3–220 mA) [140]	Small (∼200 kbps) [128]
Ambient-IoT	Long (cellular-based)	Ultra-low (0.56 μA at 1.8 V) [131]	Very small (<1 kbps) [141]
Satellite M2M/SBD	Global	Low–Moderate (34–145 mA) [142]	Very small (112–784 bps) [143]
Satellite Internet	Global	Very High (2–3 A at 12 V) [144]	Large (25–200 Mbps) [119]

Note: given the strong dependence of communication performance on deployment conditions, configuration parameters, and traffic profiles, the values reported in this table are intended to provide order-of-magnitude comparisons, often based on single commercial solution (e.g., Starlink or Iridium) rather than on universally applicable quantities.

## 5. Management and Analysis of Environmental Data

Although the infrastructures described above enable the acquisition of large volumes of environmental data, the utility of such information ultimately depends on its effective storage, management, and subsequent interpretation. Consequently, the extraction of actionable knowledge from monitored systems hinges on two fundamental aspects: the location at which data are stored and the methodologies adopted for their analysis.

Traditional approaches to environmental data analysis have largely relied on manual inspection, statistical post-processing, and offline workflows executed on local computing resources. While effective for limited datasets, such methods become increasingly impractical as monitoring systems scale in spatial extent, temporal resolution, and sensor heterogeneity [145]. As a result, the problem of data analysis is tightly coupled with that of data management and storage.

Cloud-based infrastructures address these challenges by providing centralized, scalable resources for data storage and computation [146]. Beyond simply offering a location where data can be stored, cloud platforms could enable a fundamental shift in how environmental data are handled, processed, and analyzed, supporting continuous ingestion, elastic computation, and integration across distributed sensing systems.

As a consequence, artificial intelligence (AI) and machine learning (ML) techniques have emerged as powerful tools for the automated analysis of environmental data, enabling tasks such as feature extraction, anomaly detection, classification, and prediction [147].

This chapter therefore examines the management and analysis of environmental data within cloud-based architectures, with particular emphasis on how cloud platforms act as an enabling layer for scalable data pipelines and AI-driven processing.

### 5.1. Cloud-Based Systems

In distributed environmental monitoring, cloud computing provides a centralized infrastructure for collecting, storing, and processing data from geographically dispersed measurement systems.

According to the classification proposed by the U.S. National Institute of Standards and Technology (NIST), cloud computing can be articulated into three principal service models defined by the level of access to the underlying infrastructure. In the Software as a Service (SaaS) model, users access complete applications provided by the cloud service, with control limited to application-level configuration. Platform as a Service (PaaS) offers an environment for developing and deploying custom applications while abstracting the management of the underlying infrastructure. Infrastructure as a Service (IaaS) exposes fundamental computational resources such as virtual servers, storage, and networking, providing the highest degree of control at the cost of greater operational complexity [148].

In the environmental-monitoring domain, these service models introduce distinct trade-offs in terms of flexibility, control, and responsibility in data management. SaaS solutions may simplify access to data products and analytics but limit direct intervention in data ingestion, transformation, and storage strategies. PaaS architectures could provide a balance between flexibility and abstraction, enabling the implementation of customized data pipelines while offloading infrastructure management. IaaS solutions should offer full control over computational and storage resources, supporting fully bespoke data-management workflows at the expense of increased system complexity.

These trade-offs become particularly relevant when monitoring systems generate large, continuous, multi-source, and heterogeneous data streams, as is typical of distributed environmental sensor networks. In such scenarios, effective end-to-end data lifecycle management requires structured ingestion and processing pipelines, commonly referred to as Extract, Transform, and Load (ETL) processes, sometimes extended with forecasting or advanced analytics stages [149].

Within the IoT paradigm for environmental monitoring, Big Data Management introduces additional challenges related to data heterogeneity, scalability, and processing timeliness. A persistent issue is the fragmentation of data into isolated silos, which hinders a unified interpretation of observed phenomena. To overcome these limitations, data-lake architectures—such as the CEBA system—have been introduced. These architectures leverage elastic computing stacks to ingest and manage raw data from heterogeneous sources, while providing a shared platform that facilitates data access and collaboration across the scientific community [150].

There is also a growing need to process information streams in real time rather than exclusively through batch-oriented workflows. Distributed middleware based on stream processing, for example Apache Kafka 4.2.0 decouples data production from analysis and enables low-latency computation of complex environmental indices [151].

Data management also encompasses data validation, anomaly detection, and visualization. Sensor-to-cloud platforms must ensure information quality by identifying measurement drifts and outliers in order to support reliable alerting, as demonstrated in hydrological and volcanic monitoring contexts [152]. The effectiveness of cloud-based data-management architectures is reflected in their ability to deliver real-time dashboards and decision-support tools, as shown in domains such as aquaculture, where timely access to environmental information directly affects productivity and animal welfare [153]. Table 6 summarizes the differences between the various cloud technologies discussed in this section.

### 5.2. Artificial Intelligence Techniques for Data Processing

Artificial Intelligence (AI), Machine Learning (ML), and Deep Learning (DL) techniques have emerged as key enablers for automated, data-driven analysis in environmental monitoring. These methods could allow complex patterns to be identified across large, multi-source datasets and support tasks such as feature extraction, anomaly detection, classification, and predictive modeling.

The following section describes the fundamentals of AI techniques, then surveys the application to environmental data processing.

Seeking formal definitions, Russell and Norvig characterize Artificial Intelligence as the study of agents that act rationally in response to environmental perceptions, while Machine Learning denotes the internal adaptation (training) that enables such agents to improve the effectiveness of their future actions through experience. Deep Learning is a specific form of training that employs a cascade of multiple layers of nonlinear processing to extract and transform data hierarchically [154].

Regarding the use of AI in environmental monitoring, the literature consistently links model effectiveness to the availability of large observational datasets. Cui et al., (2023) analyze the training stages of algorithms in ecological and health contexts, emphasizing the dependence of ML performance on data quantity and quality [155]. Complementarily, Olawade et al., (2024) highlight the potential of AI for analyzing large environmental datasets and generating scalable, reproducible forecasts, in contrast to traditional workflows that are often manual or semi-manual [156].

Numerous studies report ML models tailored to specific monitoring tasks. For example, Chen et al. employ Long Short-Term Memory (LSTM) networks to forecast water quality from environmental time series [157]. Other contributions apply DL to observational data for the identification and assessment of harmful algal blooms [158], or for evaluating the status of sensitive ecosystems such as coral reefs [159]. Artificial intelligence should be regarded as a pervasive analytical layer embedded within the monitoring chain, rather than as an isolated or decoupled component. When integrated into cloud-based infrastructures, AI-driven processing pipelines can systematically convert raw data into actionable knowledge through automated, scalable interpretation and predictive modeling.

Edge computing [160] is a paradigm in which substantial compute and storage resources are deployed at the Internet edge, in close physical proximity to mobile devices and sensors, rather than being confined to distant centralized cloud data centers. The choice between edge computing and cloud-side AI deployment can be justified by the coupled bandwidth–energy trade-off inherent to remote monitoring systems. In bandwidth-limited links (e.g., low-power LPWAN or satellite M2M), transmitting raw or high-rate sensor streams to the cloud may be impractical or prohibitively energy-expensive. In these settings, performing feature extraction, compression, event detection, or anomaly screening on-device reduces the communicated payload and enables sparse, duty-cycled transmissions, thereby improving energy autonomy at the cost of higher on-board computational demand and model-maintenance complexity. Conversely, when connectivity is reliable and sufficiently provisioned (e.g., coastal cellular coverage or high-throughput satellite internet), cloud-side processing becomes advantageous because it allows the use of larger models, centralized updates, richer data fusion, and reproducible pipelines, while shifting computation away from resource-constrained nodes. Accordingly, the deployment model is dictated by whether the application is constrained primarily by communication (favoring edge inference to minimize uplink volume) or by computation and maintainability (favoring cloud inference), consistent with the bandwidth and energy considerations discussed in Section 4 and Section 7.

### 5.3. Challenges and Open Issues in AI for Aquatic Monitoring

Despite the growing use of AI techniques in aquatic environmental monitoring, several structural challenges limit their robustness and operational uptake. Based on our direct research experience and the empirical evidence gathered from our ongoing real-world deployment projects, we argue that the following points summarize the most relevant challenges and open issues that currently hinder the widespread and reliable adoption of AI techniques in aquatic environmental monitoring. Our field-tested perspective suggests that these issues, spanning data availability, model generalization, interpretability, and validation under harsh environmental conditions, are not merely theoretical, but represent significant practical barriers that must be overcome to fully integrate AI within seamless, end-to-end sensing-to-cloud workflows. In more detail:

- Data scarcity and class imbalance: AI models for aquatic monitoring often rely on relatively small labelled datasets, especially for rare but critical events such as harmful algal blooms, oil spills, or extreme hypoxic episodes. This scarcity is compounded by strong class imbalance, where normal conditions dominate observational records and rare events are underrepresented, leading to biased models that may exhibit high overall accuracy yet poor sensitivity to the phenomena of greatest management interest. Approaches such as targeted field campaigns, semi-supervised learning, and synthetic data generation may help alleviate these constraints, but they cannot fully replace high-quality, event-focused observations.
- Transferability and domain shift: Models trained in a specific lake, estuary, or marine region frequently degrade when applied to other sites, even when nominally similar sensors and variables are used. This domain shift arises from differences in baseline conditions, local stressor combinations, sensor configurations, and data-quality characteristics, which alter the statistical structure of the input space and undermine out-of-domain generalization. Robust deployment therefore requires strategies such as domain adaptation, site-specific fine-tuning, or ensemble models that explicitly account for spatial heterogeneity across aquatic environments.
- Bias and interpretability for decision support: AI-based predictions inherit biases from the underlying data, including measurement artefacts, uncorrected drifts, and sampling-design constraints. Black-box models may amplify these biases while offering limited insight into the physical or biogeochemical mechanisms driving their outputs, complicating their use in regulatory or risk-management contexts where transparent justification is required. Interpretable feature representations, physically consistent architectures, and post-hoc explanation tools are therefore essential to ensure that AI-derived indicators can be critically assessed by domain experts and integrated into decision-making processes.
- Validation in real deployments: Many AI models are evaluated on historical datasets or single-site case studies, with limited evidence on their behaviour under operational conditions, evolving sensor networks, and changing environmental baselines. There is a growing need for cross-site benchmarks, standardized performance metrics, and coordinated validation campaigns that test models across multiple platforms, water types, and stressor regimes. Establishing such evaluation frameworks is a prerequisite for defining acceptance criteria and for integrating AI services into routine aquatic monitoring operations.

Taken together, these challenges point to the need for future research on risk-aware, uncertainty-conscious AI pipelines that are explicitly designed for heterogeneous aquatic contexts and validated through standardized, cross-site protocols. These aspects will be further articulated in the concluding research agenda as key open questions for next-generation aquatic monitoring systems.

## 6. Aquatic Monitoring Platforms

Throughout the preceding sections, the importance of in situ monitoring has consistently emerged as a central component for the observation and management of aquatic ecosystems. Within this framework, monitoring platforms constitute the physical layer through which sensors, data-acquisition systems, and communication technologies are integrated into the operational environment. The following analysis therefore examines the principal aquatic monitoring platforms, with particular attention to buoy-based systems and drone-enabled solutions, including surface, underwater, and aerial vehicles.

### 6.1. Buoy-Based Systems

The first platform class considered is that of buoys. These floating systems are dedicated to environmental data collection and can integrate measurement instrumentation, onboard processing, geolocation, and data transmission. Although buoys can take on different geometric configurations, in aquatic monitoring practice it is common to distinguish mainly between moored (fixed/anchored) and drifting buoys [161].

Moored buoys provide a well-established solution for continuous, long-term observations and are widely deployed in global climate-observation programs. Historical and still-operational exemplars include the Tropical Ocean Global Atmosphere (TOGA) program [162] and its subsequent PIRATA [163] and RAMA [164] arrays, designed to investigate ocean–atmosphere interactions and monsoon systems across the major tropical basins. From an architectural point of view, a moored buoy consists of a surface float held in position by a mooring on the seabed, which allows sensors to be installed both on the surface and along the water column. This configuration is particularly suitable for the continuous measurement of physical and chemical parameters, such as temperature, salinity, and pressure, often using CTD sensors distributed along the mooring line [161]. Material and construction choices depend on payload, environmental conditions, and required operational lifetime, with designs typically emphasizing robustness and long-term reliability. A representative example is the ATLAS T-Flex buoy employed in the RAMA and PIRATA programs, engineered for continuous operation in remote oceanic environments and for satellite data relay, e.g., via Iridium links [165]. Systems of this type target large-scale monitoring and therefore entail substantial requirements in terms of size, engineering complexity, and implementation and maintenance costs. Alongside these established infrastructures, a growing class of smaller-scale monitoring projects has emerged for coastal, lagoonal, and local settings. These solutions favor compact, low-power, and cost-conscious architectures, often leveraging IoT technologies and LPWAN protocols such as LoRaWAN. As an example, Majumder et al. report a moored buoy equipped with sensors for water and air temperature, pH, and salinity, powered by a photovoltaic subsystem and providing LoRaWAN connectivity to a gateway and cloud services [166]. Moored buoys can support multi-depth instrument strings (e.g., temperature/salinity and biogeochemical probes such as dissolved oxygen and chlorophyll-*a*), enabling high-quality time series with configurable sampling schedules [167].

While moored buoys enable continuous observations at a fixed, spatially defined location, drifting buoys introduce a complementary observational paradigm in which environmental sampling occurs along trajectories determined by the movement of surrounding water masses. Alongside fixed-point observatories, floating platforms remain central to global efforts in oceanographic data collection and climate research. Notable examples include the Coastal Dynamics Experiment (CODE) [168] and, in particular, the Global Drifter Program (GDP) [169], which has been instrumental in characterizing near-surface global ocean circulation. GDP Lagrangian buoys are designed to follow the flow of the water, allowing reconstruction of surface current fields and transport processes through periodic transmission of geographic position. The first generations of floating buoys from the Surface Velocity Program (SVP) were also part of the TOGA research, contributing to studies on the interaction between the ocean and the atmosphere. From a structural perspective, a standard SVP drifter consists of a spherical surface float tethered to a subsurface “holey-sock” drogue, typically at a nominal depth of approximately 15 m. This configuration is adopted to ensure that the platform’s trajectory accurately reflects the motion of the surrounding water parcel, minimizing the confounding effects of direct wind stress and wave action [161]. Lagrangian drifters are characterized by finite operational lifespans, typically governed by a median duration of approximately 94 days for SVP units [170] and generally utilize satellite telemetry for data transmission. Beyond traditional large-scale circulation studies, recent literature highlights a shift toward specialized, small-scale applications. For instance, Meyerjürgens et al. developed a compact, cost-effective drifter optimized for coastal and estuarine environments. This design prioritizes a shallow draft and logistical ease of deployment and recovery, enabling high-resolution investigations into fine-scale transport, dispersion, and the accumulation dynamics of marine debris [171]. Concurrently, technical advancements have led to the integration of reference-grade instrumentation; the Copernicus SVP-BRST project, for example, augments the baseline platform with high-accuracy temperature and pressure sensors. These additions monitor operational depth and enhance data fidelity, ultimately supporting the calibration and validation (i.e., Cal/Val) of satellite-derived sea surface temperature (SST) products [172]. Table 7 compares moored and drifting buoy characteristics.

### 6.2. Surface Vehicles (USVs and ASVs)

Unmanned Surface Vehicles (USVs) are autonomous or remotely operated marine platforms used in industrial, defense contexts and for research and environmental monitoring. Recurring USVconfigurations include catamarans, kayaks, and rigid inflatable platforms. Catamarans typically offer high stability, convenient deck access, and payload capacity suitable for heterogeneous sensor suites; kayak-based systems often derive from modified commercial hulls, reducing development time and cost; rigid inflatables are valued for structural robustness and the ability to host larger energy reserves, and are common in military deployments. Autonomous Surface Vehicles (ASVs) are distinguished within the domain of Unmanned Surface Vehicles (USVs) by their capability for autonomous navigation. Although they are frequently underactuated—that is, possessing fewer control inputs than degrees of freedom—these platforms employ sophisticated algorithms to execute missions autonomously in complex and challenging environments [173,174].

Regarding propulsion, USVs predominantly employ propellers or waterjets with rudder-based steering, although there are also sophisticated sail-powered concepts that prioritize energy efficiency [175]. In some catamarans differential thrust could be used to enhance maneuverability. A baseline sensor suite typically includes satellite positioning (GPS), wireless communication, and an inertial measurement unit (IMU); depending on mission objectives, additional payloads such as radar, sonar, cameras, and task-specific sensors are integrated [161].

USVs support a broad spectrum of applications, including environmental monitoring and sampling, disaster forecasting and response, pollution measurement and mitigation, exploratory and maintenance missions [176]. The Saildrone platform contributed to NOAA’s 2021 Atlantic Hurricane Observations Mission, collecting in-storm atmospheric and oceanic measurements [177]. Similarly, NASA deployed USVs in the S-MODE (Submesoscale Ocean Dynamics Experiment) project to investigate vortical structures and submesoscale processes [178]. Beyond massive research campaigns, the literature documents widespread use of USVs in small-scale scientific studies: for example, Jo et al. describe an open-source, low-cost USV equipped with water-quality sensors [179], while Gogendeau et al. present a compact, open-source autonomous USV designed for wildlife behavior/trajectory monitoring as well as bathymetric and photogrammetric surveys [180]. As stated in Section 7, USV energy autonomy needs to rely on comprehensive energy modeling, accounting for both static and dynamic consumption to inform simulation-based mission planning [181]. Range extension is achieved through smart energy management and solar integration, leveraging environmental forecasts to optimize trajectories [182]. The adoption of LPWAN alongside other systems (e.g., satellite) for telecommunication purposes could support efficient, low-bandwidth communication and streamlined data reporting. The use of USVs in environmental monitoring scenarios is characterized by a set of clearly identifiable operational trade-offs. Remote-controlled or autonomous surface mobility enables flexible mission planning, access to areas that are difficult or unsafe for crewed vessels, and a reduction of personnel-related risks, often accompanied by lower operational costs compared with conventional platforms [176]. These benefits are counterbalanced by inherent constraints, primarily related to limited energy autonomy, restricted payload capacity, and reliance on communication links for vehicle control and data transmission. As a consequence, USVs occupy an intermediate role between stationary observing platforms, such as fixed buoys, and fully passive or freely drifting systems. They combine controlled mobility with targeted data acquisition, without providing the long-term persistence of fixed installations or the large-scale spatial coverage of passive drifter-based observations, and could be therefore most effective when integrated within coordinated, multi-platform monitoring architectures.

### 6.3. Underwater Vehicles (ROVs and AUVs)

Unmanned Underwater Vehicles (UUVs) are crewless submersible platforms designed for operation in environments where direct human access is difficult or impractical. While their configuration is highly application dependent, these systems are generally engineered to maneuver within a fluid, regulate attitude and depth via ballast, and sustain sufficient energy autonomy to complete assigned missions. Propulsion is typically provided by one or more thrusters, sometimes complemented by hydrodynamic surfaces (fins or wings), whereas energy supply most commonly relies on batteries or external power, with advanced solutions employing fuel cells or hybrid architectures. From a communications standpoint, the underwater medium imposes stringent constraints: acoustic links are predominant, occasionally complemented by short-range optical channels or by satellite links established when the vehicle surfaces. UUVs are employed for data-acquisition missions such as bathymetric surveys, environmental monitoring of the water column and seabed, and geomorphological characterization [183].

Within the UUV class, Remotely Operated Vehicles (ROVs) are controlled in real time by a topside operator and connected to a support vessel via an umbilical (tether) that provides power, communications, and control signals. This architecture affords a high degree of control and supports complex, power-hungry payloads, making ROVs well suited to inspection, intervention, and targeted scientific tasks. Consequently, ROVs are widely used in both industrial operations and research campaigns, often by adapting commercial platforms with dedicated scientific instrumentation [184].

Autonomous Underwater Vehicles (AUVs) constitute a parallel class designed to execute missions with substantial autonomy, without a continuous link to the operator. AUVs support a broad range of applications including high-resolution bathymetry, geological exploration, oil spill detection, monitoring of dynamic phenomena (thermocline, internal waves, algal blooms), and observations of the water column and seafloor. Torpedo-like configurations are prevalent for hydrodynamic efficiency, and platforms span from portable models to large vehicles capable of operating from tens of meters to kilometer-scale depths [176].

Autonomy is both a defining feature and a principal challenge for AUVs. It does not imply the absence of human oversight; rather, it entails integrated strategies for navigation, path planning, and event response that allow the vehicle to execute preplanned missions while adapting within bounds to environmental conditions and encountered obstacles. In practice, many applications adopt intermediate autonomy levels, with the operator retaining a central role in mission planning, health/status monitoring, and vehicle recovery [185].

UUVs, including both ROVs and AUVs, are currently employed across numerous research initiatives. In Italy, for example, the MER (Marine Ecosystem Restoration) project deploys AUVs for coastal habitat mapping and ROVs for seamount exploration and characterization [186]. Globally, AUVs have also been used successfully in extreme environments such as missions beneath the Fimbul Ice Shelf in Antarctica, enabling observations in one of the least accessible regions of the world ocean [187]. Recent literature, in parallel, documents the development of small, low-cost AUVs aimed at broadening adoption in distributed monitoring campaigns and in resource-limited contexts [188,189].

Sampling strategies are frequently ‘adaptive,’ whereby the AUV dynamically modifies measurement patterns or sensor activation in response to detected environmental features. This approach aims to maximize the information gain per unit of energy expended [190]. Within the overall energy budget, the communication subsystem represents a significant factor. Specifically, the power consumption of acoustic modems can be substantial [191]. Current literature on acoustic modems, including software-defined solutions, underscores the inherent trade-off between link robustness, achievable bitrate, and the associated energy cost [192]. Relative to surface platforms, UUVs provide unique observational capabilities, namely, direct access to the seabed and deep water column, balanced by structural and operational constraints, notably energy autonomy, the complexity of navigation and communication subsystems, and the costs associated with deployment and recovery.

### 6.4. UAV Systems (Aerial Drones) for Aquatic Monitoring

Unmanned Aerial Vehicles (UAVs) comprise aircraft operated without an onboard pilot. Originating in military, industrial, and civil domains, these systems have increasingly assumed a prominent role in scientific applications owing to their operational flexibility and their capacity to acquire high-resolution data over extensive spatial domains.

Given the vast breadth of this category, no single taxonomic standard prevails. Instead, platforms are commonly categorized by size and operational flight capabilities [193]. Aerodynamically and from a propulsion standpoint, three configurations predominate: fixed-wing, multirotor, and hybrid vertical take-off and landing (VTOL). Fixed-wing UAVs feature relatively simple designs, high cruise speeds, and superior energy efficiency, making them suitable for long-range coverage, though they lack vertical take-off and hover. Multirotors, by contrast, are the most widespread and user-friendly configuration, offering vertical take-off and landing, excellent stability, and high maneuverability, at the cost of increased energy consumption. Hybrid VTOL platforms occupy an intermediate position, combining the operational flexibility of vertical take-off with flight dynamics closer to fixed-wing efficiency [194,195].

From a design perspective, UAV development is tightly constrained by weight, structural strength, and systems integration. Materials such as carbon fiber, aluminum, and engineered polymers are widely employed to balance mechanical robustness with low mass [194]. Power supply is a critical determinant of performance: endurance is directly influenced by payload weight and by the limited specific energy of batteries—one of the principal bottlenecks, particularly for small UAVs [196]. Beyond conventional lithium-ion batteries, alternative solutions have been explored, including solar-cell integration on lifting surfaces to extend operational endurance.

With respect to control and autonomy, UAVs are typically equipped with autopilots and guidance, navigation, and control (GNC) algorithms that enable execution of preplanned flight paths without continuous operator input. Nonetheless, radio-communication constraints remain significant: standard control links usually offer ranges of only a few kilometers, limiting operations in remote scenarios [195].

UAVs have been successfully deployed in major scientific missions. During MOSAiC, for example, multiple campaigns employed UAVs to characterize the thermodynamic and kinematic state of the lower atmosphere in polar environments [197]. In related contexts, these platforms have been used to monitor penguin colonies in Antarctica, reducing direct anthropogenic disturbance to observed species [198]. At more local scales, the literature documents diverse applications, including coral-reef monitoring [199] and the detection and mapping of plastic debris along coastlines [200].

UAV-assisted acquisition is also commonly implemented either as a mobile *data mule* that periodically contacts remote stations, downloads buffered packets over a short-range/LPWAN link, and uploads them to the cloud when backhaul is available, or as an aerial *relay* whose trajectory and transmission policy are optimized under energy constraints. In [201], stations package multi-sensor samples into small files (order of $10^{3}$ B), where the sampling period (e.g., tens of seconds to minutes) and the packaging interval (e.g., tens of minutes to hours) directly set the required contact time and revisit schedule. This store-and-forward model naturally promotes duty-cycled sensing, local packaging/aggregation, and brief radio transfers, while deferring bulk upload to opportunistic connectivity at the base station [201,202]. As buffered volume and airtime increase (e.g., finer sampling or richer payloads), the platform energy budget becomes a tighter constraint, motivating joint communication–trajectory design [203].

### 6.5. Cross-Layer Constraints in Sensing-To-Cloud Pipelines

As summarized in Table 8, platform constraints establish the primary design boundary for end-to-end environmental monitoring systems: fixed nodes leverage proximity to existing infrastructure, whereas drifters and long-duration deployments necessitate low-maintenance operation with conservative uplink strategies. This determines the feasible communication envelope (Table 5), defined by coverage, throughput, and duty-cycling trade-offs, which, in turn, dictate admissible upload patterns (continuous, batched, or opportunistic). Sensing and data acquisition strategies must then be adapted accordingly (Section 4): sensor modalities (scalars versus spectra/images), sampling intervals, and local data reduction techniques (aggregation, feature extraction, event-based triggering) are selected to match payload limits and latency requirements imposed by the communication link. Energy autonomy requirements complete the optimization loop (Section 7): sensor excitation, acquisition frequency, local buffering, and radio time-on-air collectively define the average power budget and requisite battery capacity or harvesting margin. In practice, this cross-layer design process iterates until platform feasibility, telemetry constraints, sensing fidelity, and energy sustainability achieve mutual consistency across the sensing-to-cloud continuum. The M3A Mediterranean multi-sensor buoy network [167] provides a clear example of how operational monitoring systems are designed by balancing measurement richness, near-real-time (NRT) availability, energy autonomy, and maintenance logistics. In the E1-M3A implementation, a modular architecture based on independent mooring lines enables more frequent servicing of the most maintenance-demanding subsystems (on the order of a few months) while keeping the surface buoy on a longer maintenance cycle, thereby reducing ship-time at the cost of higher system complexity and recovery requirements. To support data access and remote control, satellite communication (Iridium/Globalstar) is adopted, with GSM mentioned as an alternative when available, reflecting a necessity to redundance in the communication layer. High-volume payloads are not continuously streamed, since data volumes exceed practical telemetry capabilities; instead, sampling and local buffering strategies are used to preserve both autonomy and operational sustainability. E2-M3A further illustrates the classic complexity–maintainability trade-off by adopting an automatic pumped water-sampling approach that allows multi-depth biogeochemical analyses within the buoy’s protected interior, easing underwater maintenance while introducing additional mechanical subsystems. In W1-M3A, instrumentation is concentrated in the upper layer to ensure timely delivery, and the system explicitly discusses conservative effective data rates and transfer durations as drivers of operating cost and link performance, emphasizing the bandwidth–latency–cost trade-off. Finally, the hybrid energy subsystem (solar/wind plus batteries) constrains acquisition and telemetry scheduling, tying the overall monitoring strategy to low frequency (2 Hz) sensing and batched transmissions that trade immediacy for robustness and endurance.

## 7. Power Supply Systems for Energy Autonomy of Monitoring Systems

In aquatic monitoring, energy autonomy represents a critical system constraint that propagates across the entire acquisition chain. The sensing and DAQ subsystems define the baseline power budget through sensor excitation, signal conditioning, sampling, and onboard processing. In parallel, the telecommunication solutions discussed in Section 4 introduce highly variable energy costs, which often become dominant depending on payload size, duty cycle, and required coverage. Likewise, the monitoring platforms detailed in Section 6 impose distinct operational envelopes. Whereas moored buoys can accommodate larger energy reserves for extended deployments, and drifters favor simplicity at the cost of a limited operational lifespan, mobile platforms—including USVs, UUVs/AUVs, and UAVs—exhibit a more nuanced trade-off in energy management. For these assets, endurance is not only a function of the electronic payload but is primarily governed by the propulsion system. The energy demand for motion, heavily influenced by mission profiles and environmental factors (e.g., currents, drag, or wind), often constitutes the most significant drain on the onboard power supply, thereby tightly coupling controlled mobility to overall mission duration. As a result, the design of autonomous in situ monitoring systems must be framed in terms of energy availability and consumption management. Power supply architectures therefore become a critical design choice, determining feasible sampling strategies, communication schedules, payload integration, deployment duration and data continuity. The following section surveys the main approaches adopted to sustain long-term operation in aquatic environments spanning energy storage, harvesting from renewable sources, hybrid configurations, and power-aware management strategies, highlighting how they support different platform classes and monitoring objectives.

Powering remote environmental monitoring systems such as marine buoys, sensor network nodes, and autonomous platforms requires in situ energy generation solutions that overcome the limitations of traditional batteries, whose replacement is costly and logistically complex, especially in hard-to-access environments [204,205]. Consequently, a significant share of recent research focuses on energy-harvesting techniques that exploit ambient resources to ensure long-term operational autonomy [206,207].

Photovoltaic (PV) solar energy is presently the most mature and widely adopted harvesting solution for environmental monitoring in both terrestrial and marine settings [206,208]. The efficiency of PV systems installed on buoys or surface vehicles is strongly influenced by geometric and environmental factors including panel tilt, latitude, and meteorological conditions [209]. In dynamic contexts such as autonomous surface vehicles, ultralight panels could help balance energy production with hydrodynamic stability [210].

Because solar irradiance is intrinsically intermittent, energy storage is a critical component. Storage options range from lithium-ion batteries for medium-to-high loads [166] to integrated microbatteries for ultra-low-power systems that can sustain a high number of charge–discharge cycles [211].

In marine environments, wind is a complementary, high-energy-density resource, particularly attractive for continuously exposed platforms [208]. Numerous multisensor buoys integrate micro-wind turbines to power meteorological instruments and communication systems [166,212]. However, mechanical integration demands careful design, as increased windage can affect platform stability and exacerbate roll motion [212].

Conversion of the kinetic energy associated with waves and currents is an active research avenue for powering autonomous ocean devices. Proposed wave energy converters (WECs) include oscillatory mechanisms and turbines coupled to the vertical motion of waves [213,214]. Advanced concepts, such as the WEC-Glider, integrate energy conversion with vehicle propulsion, using electromagnetic generators to transform wave motion into usable electrical power [215].

At smaller scales, micro-energy-harvesting techniques based on piezoelectric materials convert wave or flow-induced vibrations into electricity, making them suitable for ultra-low-power sensors; these approaches favor compactness and simplicity, albeit with limited power density [216,217].

Thermoelectric generators (TEGs) exploit the Seebeck effect to convert temperature gradients into electrical energy [207,218]. Although their achievable power density is generally lower than that of PV systems, TEGs offer continuous generation independent of diurnal cycles. Typical applications include infrastructure monitoring such as pipelines and industrial plants where persistent thermal differences can power wireless sensors [205].

Operational experience indicates that no single harvesting technology can, on its own, guarantee reliable autonomy under all conditions. Hybrid architectures that combine multiple sources are therefore increasingly prevalent [208,212]. Such solutions reduce dependence on any single stochastic resource and enhance system resilience under variable environmental conditions [207].

Beyond generation and storage, intelligent power management is pivotal. Energy-saving strategies, including stringent sensor duty cycling and the adoption of low-power communication protocols (e.g., LPWAN), can significantly enhance node longevity [166]. Load-forecasting models and adaptive energy-management platforms further balance production, storage, and consumption in real time, improving the overall reliability of monitoring infrastructures [204,219]. Therefore, energy autonomy is a multidimensional attribute of the monitoring system. It depends on the balanced interplay between generation, storage, and management, transcending the capabilities of a single power supply technology.

## 8. Regulations for Data Collection and Processing

For environmental monitoring systems, data quality and reliability assume a central role. The objective is not merely to acquire measurements, but to produce information that can support decisions, remain comparable over time and across networks or agencies, and be defensible in technical or regulatory settings. From this perspective, data validity emerges through a comprehensive process that monitors every stage of the data lifecycle, thereby converting isolated observations into verified and reliable information.

According to guidance from European and U.S. regulators, environmental data can be considered “valid” only when produced within a formalized process that makes both their quality and intended use transparent and verifiable [220,221]. Measurement traceability constitutes a fundamental prerequisite: every result must be linked to internationally recognized references through an unbroken chain of calibrations. This requirement is essential not only for cross-comparability of measurements but also for their legal defensibility [222,223].

A further fundamental point is that no single measurement provides an exact description of reality. As discussed in Section 4, every sensor is subject to limits of accuracy and precision; the latter plays a key role in uncertainty propagation, the mathematical framework used to estimate the uncertainty associated with a final result that depends on multiple input quantities defined in the *Guide to the Expression of Uncertainty in Measurement* (GUM, JCGM 100:2008) [224]. Transforming raw data into reliable information therefore requires systematic procedures for quality control, validation, and documentation, which are necessary to assess temporal consistency and comparability with other sources. In the absence of these steps, even a technically correct measurement loses operational meaning and cannot be employed in decision-making or regulatory contexts [225].

Within the European regulatory framework, these principles find explicit application in aquatic monitoring. Directive 2009/90/EC, for example, establishes minimum performance criteria for analytical methods used to assess water status, defining specific requirements for measurement quality and reliability (Article 4) [226], while, at the same time, Directive 2000/60/EC establishes a policy framework centred on the notion of “good status” of waters and on river basin management [227]. Within the U.S. regulatory landscape, multiple instruments address data traceability, QA/QC and standardised research frameworks. In particular, EPA 40 CFR Part 136 provides the primary federal reference for standardizing analytical test procedures for pollutant measurements and prescribes stringent requirements for sampling and sample handling to support the comparability and reproducibility of results [228]. At the same time, the EPA QA/R-5 document provides the primary reference for planning and documenting QA/QC activities and for defining data acceptance criteria in environmental monitoring projects [229]. At the global level, UN-Water promotes harmonized monitoring approaches and indicator methodologies to enable consistent reporting across countries, explicitly linking monitoring design to comparability and long-term assessment objectives [230,231]. Taken together, these instruments reflect a shared institutional priority: measurements must be traceable and reproducible (through validated methods and QA/QC), while the monitoring programme must be representative (through appropriate parameter selection, sampling design, and reporting practices) in order to support credible environmental decision-making and policy evaluation.

On the other hand, data validity depends on the nominal performance of the sensor as well as on the entire acquisition, management, and control process. This systemic approach is essential if data collected in aquatic monitoring are to be used reliably for environmental assessments, for temporal and spatial comparisons, and for supporting technical and regulatory decisions at multiple scales.

In environmental monitoring systems, measurement uncertainty accumulates progressively along the full sensing-to-cloud chain—from sensor calibration errors through DAQ quantization and noise, transmission losses or compression artifacts, and finally AI-based elaboration variability. This propagation is formally described in the GUM [224] and related ISO/IEC Guide 98-3 standard [232], which provide a standardized framework for combining Type-A (statistical) and Type-B (systematic) uncertainty contributions across cascaded processing stages. The combined standard uncertainty $u_{y}$ for a final output $y=f(x_{s},x_{DAQ},x_{t},x_{AI})$—where $x_{s}$, $x_{DAQ}$, $x_{t}$, and $x_{AI}$ represent contributions from sensing, DAQ, transmission, and AI inference, respectively—follows the law of propagation of uncertainty:

(1) $$u_{y}^{2}=\sum_{i}{\left(\frac{\partial f}{\partial x_{i}}\right)}^{2}u_{x_{i}}^{2}+2\sum_{i<j}\frac{\partial f}{\partial x_{i}}\frac{\partial f}{\partial x_{j}}u_{x_{i}x_{j}},$$

where sensitivity coefficients $\frac{\partial f}{\partial x_{i}}$ weigheach stage’s contribution relative to the output, and covariance terms $u_{x_{i}x_{j}}$ account for correlations (e.g., shared thermal noise or synchronized clock drift). In practice, sensor calibration typically dominates early-stage uncertainty ($u_{s}∼$1–5%), while DAQ noise and transmission quantization amplify downstream, often requiring Monte Carlo methods for non-linear AI transformations. A key future direction involves embedding uncertainty metadata directly into the observation data model, following standards such as O&M (Observations & Measurements) extensions or UncertML schemas [233]. Each measurement would thus carry structured uncertainty estimates alongside raw values (e.g., as JSON-LD attributes: “Cvalue”: 12.34, “u”: 0.15, “unit”: “mg/L”, “covariance”: $\mathrm{mg}/L^{2}$,…) enabling downstream AI models to condition predictions on propagated uncertainties and support fully traceable, risk-aware environmental analytics.

## 9. Conclusions and Future Work

This survey provides a comprehensive review of current methodologies and technological platforms for monitoring environmental risks in aquatic ecosystems, encompassing marine, freshwater, and brackish environments. The analysis initially categorizes major stressors by water type and stressor class, thereby facilitating a systematic mapping between environmental risks and the corresponding physical, chemical, and biological parameters to be monitored. Subsequently, the survey examines the sensing-to-acquisition chain, spanning sensing technologies, data acquisition systems, and telecommunication infrastructures. It highlights that no single communication paradigm can universally satisfy all monitoring requirements; instead, the selection of communication solutions is inherently application-specific, necessitating trade-offs among coverage, throughput, latency, energy efficiency, and infrastructure control.

The management and interpretation of large-scale environmental datasets are also discussed, emphasizing cloud-based infrastructures as scalable resources for data storage and computational processing. Artificial intelligence and machine learning techniques are framed as critical enablers for automated feature extraction, anomaly detection, classification, and predictive modeling within end-to-end sensor-to-cloud workflows. Furthermore, the survey analyzes key monitoring platforms—including buoy-based systems, as well as unmanned surface, underwater, and aerial vehicles—representing the operational layer where sensing, processing, geolocation, and communication capabilities are integrated under real-world constraints.

Finally, the review underscores that energy autonomy is a systemic characteristic of monitoring systems, arising from the coherent integration of energy harvesting, storage solutions, and power-aware management strategies, rather than from the selection of a single power source. Concurrently, it emphasizes that data validity and regulatory compliance require a structured lifecycle approach, encompassing measurement traceability, uncertainty-aware methodologies, and rigorous procedures for quality control, validation, and documentation. Collectively, the proposed end-to-end perspective offers a reference framework for designing aquatic monitoring workflows that align risk drivers, measurement requirements, technological choices, and data-quality constraints.

### Future Research Agenda: Open Research Questions

To further integrate the proposed end-to-end *risk-to-system* lens, the following open research questions (RQs) link the survey dimensions covered in Section 3, Section 4, Section 5, Section 6, Section 7 and Section 8 into a coherent research agenda:

1. RQ1—How can risk-based design principles be formalized to the extent of mapping particular stressor profiles to sensing–comms–platform designs?
  This question extends the stressor-to-parameter mapping (Section 3) by linking it to the technology alternatives discussed for sensing, DAQ and communications (Section 4) and platform integration (Section 6). The central challenge is to translate qualitative risk narratives into operational, repeatable design policies (e.g., selecting sensor families, sampling/DAQ configurations, communication links, and deployment infrastructures) while making the resulting trade-offs explicit.
2. RQ2—What standardised measures and benchmarks are needed to compare DAQ and telecommunication options in realistic conditions of aquatic environments?
  In spite of the sensing and communication design space outlined in Section 4. Comparability remains very low without common metrics and benchmark protocols that can reflect aquatic constraints (e.g., biofouling, salinity/temperature variability, channel intermittency, and infrastructure availability). The issue is to specify benchmarks that would simultaneously be able to supportdata quality, link performance and energy per delivered bit in the field conditions.
3. RQ3—How can measurement and model uncertainty be quantified and propagated throughout the entire sensing-to-cloud chain?
  Uncertainty-conscious sensing, processing, and analytics are encouraged in Section 4, Section 5 and Section 6, and traceability and data-quality requirements are contextualized in Section 8. This is encouraged by open challenges: (i) reporting measurement uncertainty uniformly across heterogeneous sensors and DAQ pipelines; (ii) propagating uncertainty via preprocessing, fusion, machine learning-based inference; and (iii) reporting uncertain metadata across the edge-to-cloud continuum in a manner such that the resulting downstream products (maps, alarms, forecasts) are auditable and decision-relevant.
4. RQ4—What validation procedures do we need to deploy AI models in a heterogeneous aquatic environment?
  Section 5 emphasizes the importance of AI/ML in the end-to-end processes, yet reliable deployment requires assurance procedures that captures changes in the domain in terms of location, time of year, and water composition. The difficulty is to set up protocols to include (i) sound ground-truthing techniques, (ii) cross-site and cross-season testing, (iii) failure-mode reporting (e.g., false alarms vs missed events) and (iv) governance aspects aligned with the data lifecycle and compliance debate in Section 8.
5. RQ5—How do we co-design multi-platform structures (buoys, USVs, UUVs, UAVs) to achieve purposeful use of multi-platform coverage, resolution and energy utilisation?
  Section 6 investigates platform families, whereas Section 4 (sensors, DAQ and communications) and Section 7 (energy autonomy) constrains the feasible operations. Open challenges include: co-optimizing (i) spatial/temporal coverage and adaptive resolution, (ii) cooperative scheduling and task allocation, (iii) cross-platform networking and data offloading, and (iv) energy-aware mission planning to ensure that multi-platform systems act as a coordinated monitoring architecture, as opposed to independent assets.

In conclusion, addressing these Research Questions is not merely a technical necessity but a fundamental requirement for transitioning from experimental monitoring to robust, operational environmental surveillance. By overcoming the current fragmentation across the sensing-to-cloud pipeline, the research community can provide the reliable, real-time data needed to safeguard aquatic ecosystems and support evidence-based policy-making on a global scale.

## Figures and Tables

**Table 1:** Comparative representation of key aspects across related works.

Key Aspects	[6]	[7]	[8]	[9]	[10]	[11]	[12]	[13]	Proposed
Aquatic Stressors	●	●	●	●					●
Autonomous Platforms		●	●	●				●	●
Biological Indicators	●	●							●
Cloud & AI Analysis	●			●	●	●	●	●	●
Digital Infrastructure					●	●	●	●	●
Energy Harvesting					●	●	●		●
Internet of Underwater Things						●			
Metrology & Standards	●	●	●	●				●	●
Remote Sensing				●					
Sensor Node Networks							●		

Note: the symbol ● indicates that the specific aspect is significantly addressed or integrated within the referenced study.

**Table 2:** Conceptual classification of major environmental stressors in aquatic ecosystems according to the type of observable alteration they induce.

Water Type	Environmental Stressor	Type of Observable Alteration
Marine	Micro/nanoplastics	Presence of suspended particulate matter
	Macroplastics	Presence of suspended particulate matter
	Oil and chemical spills	Presence of suspended chemical phases
	Eutrophication and HABs	Presence of dissolved chemical substances; alteration of biological indicators
	Underwater noise pollution	Alteration of physical energy fields (acoustic)
	Overfishing	Alteration of biological or ecological indicators
	Ocean warming	Alteration of physical properties (temperature)
	Ocean acidification	Alteration of physical properties (pH); presence of dissolved chemical substances
	Sea-level rise	Alteration of hydrodynamic or geomorphological processes
Freshwater	Nutrient enrichment and HABs	Presence of dissolved chemical substances; alteration of biological indicators
	Emerging contaminants	Presence of dissolved chemical substances
	Industrial and agricultural effluents	Presence of dissolved and suspended chemical substances
	Hydraulic works (dams, channelization)	Alteration of hydrodynamic or geomorphological processes
	Flood events	Alteration of hydrodynamic processes
	Drought conditions	Alteration of hydrological regime and physical properties
Brackish	Multistressor contamination	Combined alteration of physical, chemical, and biological indicators
	Legacy contamination	Presence of dissolved chemical substances; sediment-associated alterations
	Metal–microbiome interactions	Alteration of biological indicators linked to chemical contamination
	Climate change impacts	Alteration of physical properties and hydrodynamic processes

Note: the proposed classification is intended as a conceptual framework that groups environmental stressors according to the type of observable alteration they induce in aquatic systems, without prescribing specific monitoring parameters or measurement techniques.

**Table 3:** Exemplary mapping between selected aquatic environmental risks and monitoring system design choices along the sensing-to-cloud chain.

Risk Section 3	Key Observables Section 3.4	Sensing & DAQ Section 4	Comm. Options Section 4	Platforms Section 6
Plastics	Micro/nanoplastics size and polymer proxies; floating-litter density; drift pathways	Surface sampling (manta, pumping); imaging/vision for macro-litter; GPS drifters; low-power logging	LPWAN for status; cellular or satellite backhaul for images/summaries	Drifters; fixed nodes; surveys with USVs and UAVs
Oil & Spills	Hydrocarbon fluorescence/absorption; slick extent; thickness proxies; evolution	UV–Vis absorption and fluorescence; high-rate “burst” acquisition; active optical sensing	Satellite M2M (offshore); cellular (near coast); LPWAN for alarms/status	Moored sentinel buoys; rapid surveys with USVs/UAVs
HABs	Chlorophyll-*a*; nitrogen and phosphorus; dissolved oxygen; turbidity; temperature	Multiparameter sondes; wet-chemistry analyzers; low-power DAQ with duty-cycling	LPWAN or NB-IoT; Wi-Fi/Ethernet at fixed sites; cellular backhaul	Moored buoys; fixed river/shore stations; USV profiling
Effluents	Conductivity; nutrients; turbidity; optical proxies (BOD/COD); pesticides; flow context	Electrochemical and UV–Vis spectroscopy; passive or spot sampling; low-power loggers	Cellular (4G/5G, NB-IoT); LPWAN (rural); satellite (remote)	River/bridge stations; near-outfall nodes; small USVs
Climate	SST and subsurface structure; sea level; surge; land motion (GNSS); salinity intrusion	Temperature chains/CTD; pressure sensors + GNSS; ADCP; salinity sensors	Satellite for remote arrays; cellular/LPWAN for coastal; opportunistic upload	Large moored arrays; coastal stations; UUVs/AUVs missions
Brackish	Temp, S, pH, DO; Chl-*a*; nutrients; turbidity; sediment quality indicators	Multiparameter sondes; passive samplers; biosensors; low-power data acquisition	Cellular or LPWAN; satellite backhaul for remote deltas and lagoons	Moored lagoon stations; drifters; USV transects

**Table 6:** Conceptual comparison of cloud service models for environmental monitoring systems.

Model	User Control	Complexity	Typical Role in Environmental Monitoring
SaaS	Low	Low	Data visualization, reporting, and access to pre-defined analytics tools
PaaS	Medium	Medium	Implementation of custom ETL pipelines, data analytics, and application-level services
IaaS	High	High	Fully customized, large-scale data management and analysis architectures

Note: the table highlights conceptual trade-offs among cloud service models rather than implementation-specific details. Actual choices depend on data volume, system complexity, and long-term operational requirements.

**Table 7:** Operational comparison between Moored and Drifting buoys.

Feature	Moored Buoys	Drifting Buoys (Lagrangian)
Positioning	Fixed (anchored to the seabed)	Mobile (trajectories via water mass)
Primary Goal	Continuous, long-term observation	Surface current and transport studies
Architecture	Surface float + mooring line	Surface float + “holey-sock” drogue
Target Depth	Surface and along water column	Surface with submerged drogue at ∼15 m
Operational Life	Long-term/multi-year reliability	Finite (e.g., ∼94 days for SVP)
Connectivity	Satellite, LPWAN	Satellite telemetry

Note: configuration parameters and operational lifespans are based on established programs (e.g., RAMA, PIRATA, GDP) and technical literature cited in the text.

**Table 8:** Platform-level strengths and limitations for aquatic monitoring.

Platform	Strengths	Weaknesses/Constraints
Moored buoys	Multi-month to multi-year persistence for long-term time series Stable reference point for inter-comparisons and Cal/Val Payload and energy budget can exceed most mobile platforms	Spatially fixed (limited coverage of gradients) Biofouling and exposure drive servicing and operational costs Mooring/anchor risks and logistics (deployment/recovery)
Drifting buoys	Sampling with wide spatial coverage Long endurance with low-power payloads Scalable deployments for synoptic observations	Uncontrollable spatial coverage Limited payload/energy Typically limited sensor-wise Loss/recovery uncertainty and exposure-driven failures
USVs/ASVs	Controllable spatial coverage Flexible mission planning Reduced crew risk Rapid retasking Supports heterogeneous sensor suites during transects	Propulsion is a dominant energy sink Sea-state and collision/traffic constraints Payload and energy endurance remain limited vs fixed stations
UUVs	Access to deep water column, seabed, and under-ice environments High-resolution mapping and close-proximity sensing Minimal surface detectability for specific missions	Navigation and recovery are complex and operationally costly Underwater communication is severely bandwidth-limited Mission risk increases with duration and environment severity
UAVs	Rapid deployment and very high spatial resolution Flexible revisit scheduling for targeted surveys Effective for optical/IR mapping and shoreline reconnaissance	Short endurance Strict payload/weight constraints Weather sensitivity and regulatory limitations

Note: this qualitative comparison is complemented by platform-specific sampling/telecommunication considerations discussed in Section 4 and Section 7.

## Data Availability

No new data were created or analyzed in this study. Data sharing is not applicable to this article as all supporting data are provided within the cited references.
